# Demosaicing of RGBW Color Filter Array Based on Rank Minimization with Colorization Constraint

**DOI:** 10.3390/s20164458

**Published:** 2020-08-10

**Authors:** Hansol Kim, Sukho Lee, Moon Gi Kang

**Affiliations:** 1 School of Electrical and Electronic Engineering, Yonsei University, Seoul 03722, Korea; solchhs@yonsei.ac.kr; 2 Division of Computer Engineering, Dongseo University, Busan 47011, Korea; petrasuk@gmail.com

**Keywords:** RGB-White, color filter array, color interpolation, rank minimization, low light condition

## Abstract

Recently, the white (w) channel has been incorporated in various forms into color filter arrays (CFAs). The advantage of using theWchannel is thatWpixels have less noise than red (R), green (G), or blue (B) (RGB) pixels; therefore, under low-light conditions, pixels with high fidelity can be obtained. However, RGBW CFAs normally suffer from spatial resolution degradation due to a smaller number of color pixels than in RGB CFAs. Therefore, even though the reconstructed colors have higher sensitivity, which results in larger Color Peak Signal-to-Noise Ratio (CPSNR) values, there are some color aliasing artifacts due to a low resolution. In this paper, we propose a rank minimization-based color interpolation method with a colorization constraint for the RGBW format with a large number ofWpixels. The rank minimization can achieve a broad interpolation and preserve the structure in the image, and it thereby eliminates the color artifacts. However, the colors fade from this global process. Therefore, we further incorporate a colorization constraint into the rank minimization process for the better reproduction of the colors. The experimental results show that the images can be reconstructed well, even from noisy pattern images obtained under low-light conditions.

## 1. Introduction

An image sensor consists of a two-dimensional (2-D) array of photodiodes, where the number of photons absorbed by a photodiode determines the brightness value of the pixel at that position. However, the color of the photon, i.e., the wavelength of the photon cannot be discriminated by the photodiode. Therefore, in order to discriminate the color, a small filter that receives either red (R), green (G), or blue (B) (RGB) spectrum is coated in front of each pixel, and the arrangement of the different types of filter is called the color filter array (CFA). Various types of CFAs have been designed to sample the RGB pixels from a single sensor array starting with the widely used Bayer pattern [1], and color interpolation methods for these patterned images have also been developed [2,3,4,5,6,7,8,9,10,11,12,13,14,15]. If the input is a sequence of images, i.e., a video, the temporal information can be incorporated into the joint demosaicking and denoising task [16,17].

Recently, the demand for high-sensitivity color images has increased for various products such as autonomous driving cars and surveillance cameras.Multispectral filter arrays have been recently proposed to capture extra information other than the three primary color channels to overcome the physical limitations of the RGB CFA. For example, near-infrared pixels have been incorporated into the RGB CFA, which results in the RGB-NIR CFA, to increase the contrast between objects in the scene and the optical depth [18,19,20,21,22,23,24]. However, normally, the color images reconstructed from RGB-NIR CFAs have lower SNR values than those reconstructed from RGB CFAs, as the correlation of the brightness values of the near-infrared pixels and those of the RGB pixels are not so strong. To increase the SNR value in the reconstructed color image, a white (W) channel has been proposed to be used together with RGB channels, as the W channel can absorb more photons than RGB channels because it absorbs the full spectrum of the visible light [25,26]. The advantage of using the W channel is that W pixels have less noise than RGB pixels; thus, under low-light conditions, pixels with high fidelity can be obtained. Furthermore, the correlation of the W pixels to the RGB pixels is stronger than the near-infrared pixels.

Various types of RGBW CFAs [27,28,29,30] and their corresponding interpolation methods [25,26,31,32,33] have been proposed to reproduce a color image with high SNR values and high fidelity to the original colors. Especially, Kim and Kang have proposed an adaptive demosaicing method for the Sony RGBW CFA, where the reconstruction of the W channel is preferred to the reconstruction of the color difference channels to overcome the lack in the color information [25], while Rafinazari and Dubois have proposed a demosaicking algorithm for the Kodak RGBW CFA by reducing the overlap between the luma and chroma components. Tian et al. proposed a method that automates the design of image processing pipelines for novel color filter arrays due to the difficulty in applying appropriate image processing methods on novel color filter arrays [34]. In all these conventional interpolation techniques, a local interpolation is usually performed, which take pixels of the neighborhood into account. As W pixels have no color information, normally, the color interpolation process becomes more complex than using the conventional Bayer CFA. The additional use of W pixels also reduces the density of the RGB pixels making the color interpolation more difficult and complex. Therefore, previously, we tried to improve the sensitivity and the resolution in the RGBW CFA before applying demosaicing on the RGBW CFA [35]. However, the difficulty increases if noise is present in the sensed pattern image as the noise is spread out to neighboring pixels by the interpolation. The noise does not follow an ordinary Gaussian distribution, but it shows blob-like structures of low frequency, also called color bleeding. Therefore, the number of W pixels is usually lower than the total number of RGB pixels in conventional RGBW CFAs.

In this paper, we propose a rank minimization-based matrix completion algorithm with a colorization constraint, which can be regarded as a global color interpolation with a local constraint. The rank minimization-based matrix completion reduces color bleeding artifacts that appear in local interpolation. This is because the matrix completion takes the overall structure in the image into account and it attempts to obtain a low-rank structure, thereby reducing local artifacts. The matrix completion works well with low-rank images even in the case of little information. This fact favors the use of W-dominant CFAs [36], which have more W pixels than RGB pixels. Therefore, we first interpolate W pixels using a conventional local interpolation method, which is an easy task owing to the large number of W pixels. After that, we reconstruct the remaining R–W, G–W, and B–W channels while using the proposed method. These difference channels (hereafter called color difference channels) are of low rank by nature and, thus, can be well reconstructed, even though the numbers of RGB pixels in W-dominant CFAs are small. Furthermore, the low-rank property of the matrix completion effectively removes the noise. However, the colors slightly fade from the global low-rank interpolation. Therefore, we incorporate the colorization-based constraint into the rank minimization process for a better reproduction of the colors in the reconstructed color image. The rank minimization and the colorization mutually constrain each other and they are iteratively and alternatingly applied on the image being reconstructed. At the end of the iteration, i.e., at the convergence state, the resulting image becomes the desired reconstructed color image. The experimental results show that the proposed method can produce a reconstructed image of high visual quality, even in the presence of noise.

## 2. Related Works

In this section, we explain the performed work related to the proposed method.

### 2.1. Levin’s Colorization

Levin’s colorization algorithm [37] has been introduced as an algorithm that can colorize a gray image with a small number of color pixels. Let *M* be the number of pixels in the color image, and y and u be the vectorized luminance and chrominance images of size M×1. Levin’s colorization attempts to find u given y and a small number of color seeds. Let x denote an M×1 vector that contains these color seeds, i.e., the chrominance values only at the positions that belong to Ψ and zeros at all other positions. Here, Ψ denotes the set of positions of the color seeds in x, i.e., Ψ is the set of all *r* where x(r)≠0, and *r* denotes the pixel position index in raster-scan order (1≤r≤M). The colorization is performed by minimizing the following functional J(u):

(1) $$\begin{array}{c} \begin{array}{c} J\left(u\right)={∥x-Au∥}^{2}, \end{array} \end{array}$$

where A=I−W, I is an M×M identity matrix, and W is a weighting matrix of size M×M defined as

(2) $$\begin{array}{c} \begin{array}{c} W\left(r,s\right)=\left\{\begin{array}{c} \omega_{rs}\;\;\;\mathrm{if}r\notin \Psi \mathrm{and}s\in N(r)\\ 0\;\;\;\mathrm{otherwise}, \end{array}\right. \end{array} \end{array}$$

where N(r) is the set of the 8-neighborhood pixels of *r*. The weighting coefficient $\omega_{rs}$ is designed as

(3) $$\begin{array}{c} \omega_{rs}\propto e^{-{\left(y\left(r\right)-y\left(s\right)\right)}^{2}/2\sigma_{r}^{2}}, \end{array}$$

where $\sigma_{r}^{2}$ is a positive number. The minimizer u of J(u) is the reconstructed chrominance channel, which, together with y can be composed into the reconstructed color image. The colorization technique brought about the idea that RGB channels can be reconstructed using a small number of sensed color pixels that contain the true color information and a full-resolution W channel.

### 2.2. Colorization Based Color Interpolation

Previously, we proposed a W-dominant RGBW CFA, where 75% of the sensor area is composed of W pixels [36]. Using the W-dominant RGBW CFA, the interpolation of the W channel becomes an easy task, and the interpolation can be performed using any local interpolation technique. After the W channel is fully interpolated, the color channels can be obtained while using the aforementioned colorization scheme. Let w and c represent the lexicographically ordered vectors corresponding to the W channel and one of the RGB color channels, i.e., c∈{r,g,b}. Subsequently, the color difference channel $u_{c}$ to be reconstructed is defined as:

(4) $$\begin{array}{c} \begin{array}{c} u_{c}=c-w. \end{array} \end{array}$$

As the pattern only samples about 25/3≈8% of the components for each color channel c, the input vector $x_{c}$ is a sparse vector containing chrominance values only at the positions where the colors are sensed, and zero values at all other positions:

(5) $$\begin{array}{c} \begin{array}{c} x_{c}\left(m\right)=\left\{\begin{array}{cc} c\left(m\right)-\hat{w}\left(m\right) & \mathrm{if}m\in \Psi_{c}\\ 0 & \mathrm{otherwise}, \end{array}\right. \end{array} \end{array}$$

where $\hat{w}\left(m\right)$ is the reconstructed W pixel at $m\in \Psi_{c}$, and $\Psi_{c}$ represents the set of the pixel positions where the colors of c are sensed by the RGBW CFA. The white channel $\hat{w}$ is reconstructed by an 8-neighborhood weighted interpolation,

(6) $$\begin{array}{c} \hat{w}\left(m\right)=\frac{\sum_{n\in N(m)}\alpha_{n}w\left(n\right)}{\sum_{n\in N(m)}\alpha_{n}}, \end{array}$$

where $\alpha_{n}$ is a directional weighting parameter and N(m) denotes the eight-neighborhood of *m*. In the experiments, we used the same directional weights as in the method of Paul et al. [36], but any other directional interpolation will also give good results, since the ratio of white pixels is 75% of the whole CFA domain.

## 3. Proposed Method

**Figure 1:**
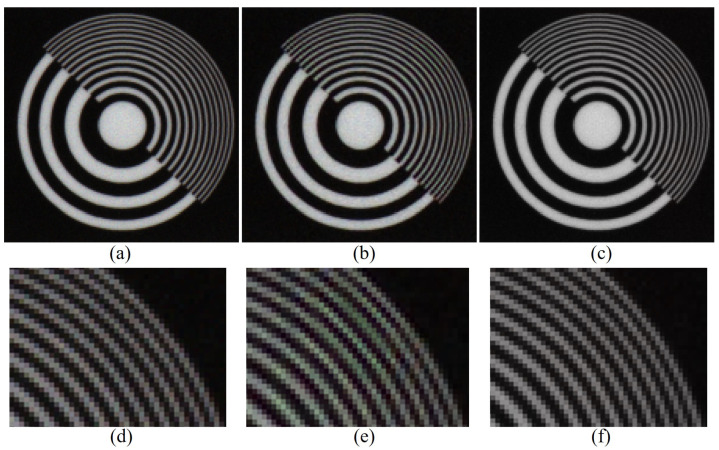
Effect of the rank minimization-based global interpolation, as described in (7). Experimental results on a partially cropped photo image of the ISO 12233 Resolution Chart. (**a**) Original; (**b**) Reconstructed using residual interpolation [14]; (**c**) Reconstructed by (7); (**d**) Enlarged region of (**a**); (**e**) Enlarged region of (**b**); (**f**) Enlarged region of (**c**).

In this section, we propose an iterative rank-minimization-based matrix completion method with a colorization constraint for filling in the missing pixels, i.e., for demosaicing of W-dominant RGBW random-pattern images [36]. Previously, a matrix completion-based interpolation method has been proposed for homogeneous autofluorescence hyperspectral images [38]. However, to our knowledge, it has not been used for demosaicing of normal images, because matrix completion usually works well with low-rank images, but not with normal images. However, for a W-dominant RGBW pattern image, the matrix completion can be a good match owing to the following facts. First, the main problem with W-dominant RGBW pattern images is the reconstruction of color difference channels, i.e., R–W, G–W, and B–W difference channels, which have low ranks and, therefore, can be well reconstructed using the minimum-rank matrix completion. Second, the W channel, which contains high-frequency components, can be easily reconstructed while using any type of local interpolation method as W pixels cover 75% of the whole CFA. Third, the minimum-rank matrix completion works well with random patterns. Normally, with CFAs which have periodical patterns, the contributions of pixels to the directional interpolation of a certain color are different for different positions, which results in the aliasing artifact. However, with the random CFA, the contributions of pixels of different colors to the interpolation of a certain color are almost the same at every position and, therefore, the aliasing artifact is reduced to some extent. However, as a random pattern is also unstructured, the colors are interpolated in an unstructured local way using local interpolation techniques, which leads to color permeation. The rank minimization technique, which takes the global structure of the image into account, can overcome this problem to some extent. The constrained rank minimization based demosaicing problem can be formulated as:

(7) $$\begin{array}{c} \begin{array}{cc} \min_{U_{c}} & {\mathrm{rank}}_{DCT}\left(U_{c}\right)\\ \;\mathrm{subject}\;\mathrm{to}\; & U_{c}\left(r\right)=X_{c}\left(r\right)\;\forall r\in \Psi_{c}, \end{array} \end{array}$$

where ${\mathrm{rank}}_{DCT}$ denotes the fact that we minimize the rank with respect to the discrete cosine transform basis, and $U_{c}$ is the color difference channel in a 2-D image form and Xc is the 2-D matrix containing the color difference pixels at the positions where R (or G/B) pixels are sensed:

(8) $$\begin{array}{c} X_{c}\left(r\right)=\left\{\begin{array}{cc} C\left(r\right)-\hat{W}\left(r\right) & \mathrm{if}r\in \Psi_{c}\\ 0 & \mathrm{otherwise}, \end{array}\right. \end{array}$$

where r is the 2-D position vector, C is the sensed R (or G/B) pattern image, $\hat{W}$ is the reconstructed W channel, and $\Psi_{c}$ is the set of r at which pixels of a specific color (c=r, c=g, or c=b) are sensed, i.e., $\Psi_{c}$ can be either $\Psi_{c=r}$, $\Psi_{c=g}$, or $\Psi_{c=b}$ corresponding to the color difference channel ($U_{c=r-w}$, $U_{c=g-w}$, or $U_{c=b-w}$) we want to reconstruct. The constraint in (7) that $U_{c}\left(r\right)=X_{c}\left(r\right)$ for $\forall r\in \Psi_{c}$ keeps the sensed color values intact when minimizing $\mathrm{rank}(U_{c})$. Note that the color difference channel cannot be treated in the vectorized one-dimensional (1-D) form as a 1-D vector has a rank of one; therefore, $U_{c}$ is 2-D. As can be seen in Figure 1, the rank minimization-based global interpolation can achieve structured results as it takes the global structure into account. This reduces the aliasing artifact and false-color artifacts, which can be observed in Figure 1b,e, which is reconstructed using the residual interpolation (RI) method [14], a representative of local interpolation methods.

**Figure 2:**
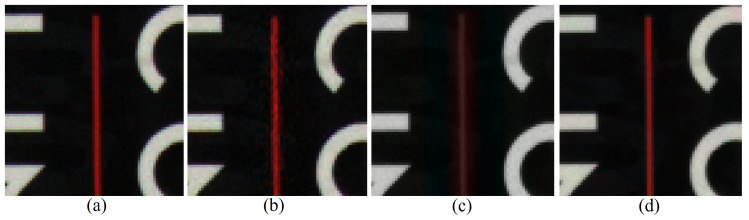
Effect of the rank minimization-based global interpolation with a colorization constraint, as described in (9). Experimental results on a photo image of a book. (**a**) Original; (**b**) Reconstructed using residual interpolation [14]; (**c**) Reconstructed by (7); (**d**) Reconstructed by (9).

However, although the rank minimization reduces the local artifacts in the structures of the image, it also results in the global smoothing of the colors, which leads to color fading, i.e., less-saturated colors. This can be observed in Figure 2. The red line in Figure 2b reconstructed using the RI method shows some local artifacts. The rank minimization-based global interpolation achieves a more structured result, i.e., removes the local artifacts, but the color fades, as can be seen in Figure 2c. This is due to the fact that the fading of the red line reduces the rank and, therefore, the attempts to minimize the rank fade the red line. To handle these issues, we solve the demosaicing problem by the following minimization problem:

(9) $$\begin{array}{c} \begin{array}{cc} \min_{U_{c}} & {\mathrm{rank}}_{DCT}\left(U_{c}\right)+\lambda J\left(\mathrm{vec}\left(U_{c}\right)\right)\\ \;\mathrm{subject}\;\mathrm{to}\; & U_{c}\left(i,j\right)=X_{c}\left(i,j\right)\;\forall r\in \Psi_{c}. \end{array} \end{array}$$

Here, λ is a small positive value that controls the balance between the rank minimization term and the colorization-based constraint, and $J(\mathrm{vec}\left(U_{c}\right))$ is the colorization-based constraint term defined as

(10) $$\begin{array}{c} J\left(\mathrm{vec}\left(U_{c}\right)\right)={∥A_{c}\mathrm{vec}\left(U_{c}\right)-\mathrm{vec}\left(X_{c}\right)∥}^{2}, \end{array}$$

where vec(M) denotes the vectorization operator that vectorizes a 2-D matrix M into a 1-D vector, and $A_{c}\in R^{M\times M}$ is defined as follows:

(11) $$\begin{array}{c} A_{c}\left(r,s\right)=\left\{\begin{array}{ccc} 1+\gamma & \mathrm{if}r\in \Psi_{c}\mathrm{and}s=r & \left(11.1\right)\\ -\gamma \omega_{rs} & \mathrm{if}r\in \Psi_{c}\mathrm{and}s\in N\left(r\right) & \left(11.2\right)\\ 1 & \mathrm{if}r\notin \Psi_{c}\mathrm{and}s=r & \left(11.3\right)\\ -\omega_{rs} & \mathrm{if}r\notin \Psi_{c}\mathrm{and}s\in N\left(r\right) & \left(11.4\right)\\ 0 & \mathrm{otherwise}. & \left(11.5\right) \end{array}\right. \end{array}$$

where *r* and *s* are 1-D indices corresponding to the 1-D positions of pixels in the vector $\mathrm{vec}(U_{c})$, and N(r) denotes the set of 1-D indices that correspond to the 2-D neighborhood of the pixel corresponding to *r*, and γ is a small positive value which decides the amount of diffusion at the color seeds. In the experiments, we let λ=0.1 and γ=0.037. Finally, $\omega_{rs}$ is the weight function computed from the reconstructed W channel $\hat{w}$ defined as

(12) $$\begin{array}{c} \omega_{rs}\propto e^{-{\left(\hat{w}\left(r\right)-\hat{w}\left(s\right)\right)}^{2}/2\sigma_{r}^{2}}, \end{array}$$

and $\sigma_{r}^{2}$ is a positive number. The constraint in (10) is a simplified version of the functional used in [36], where it is used together with a weighting kernel. Here, we use it as a constraint term to overcome the color fading artifact of the rank minimization. The local diffusion, which reduces the global color fading artifact, is performed by minimizing the energy functional in (10). The elements in matrix $A_{c}$ as defined in (11) determine the amount of local diffusion. The condition in (11.1) and (11.2) together define the amount of diffusion at the color seeds ($r\in \Psi_{c}$) and at their neighborhoods. If γ=0, there is no diffusion at the color seeds, and the color seeds are preserved as they are sensed. However, in this case, the noise in the color seeds will propagate to neighboring pixels by the local diffusion. Therefore, a small diffusion in the color seeds is controlled by a small positive value of γ and $\omega_{rs}$ to remove the noise in the color seeds. Meanwhile, (11.3) and (11.4) account for the diffusion of pixels other than the color seeds, i.e., for $r\notin \Psi_{c}$, where the amount of diffusion is determined by $\omega_{rs}$.

Figure 2d shows the result of the proposed method, i.e., the result of applying the minimization in (9). As can be seen, both the global structure and the color of the red line are well preserved. Even though the attempt to minimize the rank fade the red line, the minimization of the colorization constraint keeps and diffuses the colors of the color seeds. Consequently, the red line is reconstructed with a low-rank structure, but maintaining its colors, i.e., Figure 2d is the result of the trade-off between these two conflicting minimization processes. Compared with Figure 2b, the local artifacts are reduced in Figure 2d, whereas the colors are better preserved than in Figure 2c. When applied on a noisy CFA image, the low-rank minimization will act as a simultaneous demosaicing and denoising process, whereas the colorization constraint will preserve the colors and act as an additional demosaicing process guided by the W channel.

**Figure 3:**
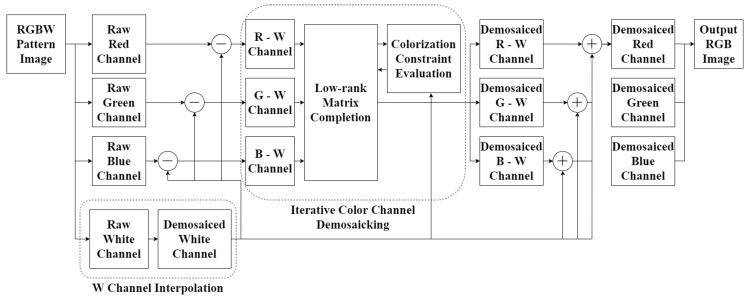
Diagram of the proposed method.

Figure 3 shows the overall diagram and Algorithm 1 shows the detailed algorithm of the proposed method. The rank minimization-based matrix completion and the colorization constraint evaluation are iteratively and alternatingly performed. The rank minimization-based matrix completion can be performed by various methods. Here, we use the simplest approach that is based on the use of a fixed basis, i.e., the patch-based DCT(discrete cosine transform) basis. First, we decompose the whole image into local patches and vectorize them. The vectorized local patches are composed into a matrix as column vectors. Subsequently, we decompose this matrix by the DCT transform and reduce the rank by reducing the number of non-zero coefficient values. The matrix completion is performed by multiplying the non-zero coefficient values with their corresponding DCT basis and composing them together. The colorization constraint evaluation is performed using the conjugate gradient method as described in Algorithm 1, and the result of the matrix completion process is updated. After that, again, the rank minimization-based matrix completion is applied on the resulted image. The whole process is iterated until the maximum iteration is reached.
**Algorithm 1.** Algorithm of the proposed method.
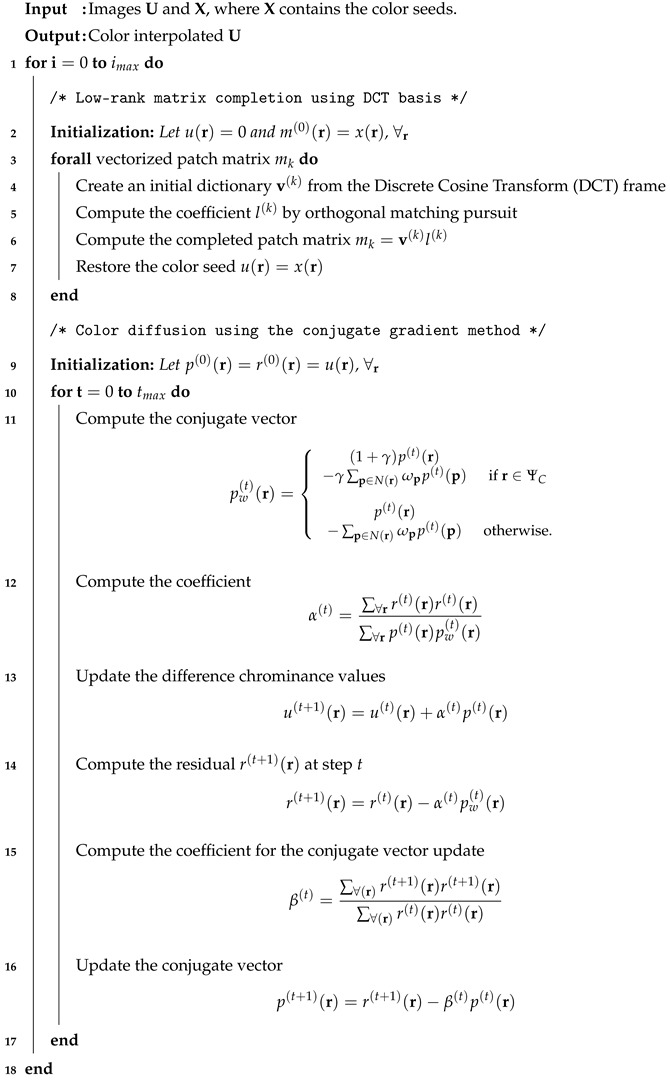



## 4. Experimental Results

We compared the proposed method with three different CFA patterns, i.e., the Bayer CFA as a representative of RGB CFAs, Sony CFA as a representative of RGBW CFAs with 50% W pixels, and the W-dominant CFA, which has 75% of W pixels and the remaining 25% of the pixels are equally distributed as RGB pixels. We performed experiments for two different cases: low noise and high noise. The noise was added to the RGB channels, and then the channels were sampled according to the different CFA patterns. In the low-noise case, the noise is derived from a zero-mean Gaussian distribution with standard deviations of 0.0463, 0.0294, 0.0322, and 0.0157 for the R, G, B, and W channels, respectively. The amount of noise is different for the R, G, B, and W channels as the different color filters absorb different light energies. The ratio between the standard deviations of the noises in the R, G, B, and W channels correspond to real physical measurements with real sensors. Here, we simulated the noise on noiseless datasets in order to calculate the CPSNR (Color Peak Signal-to-Noise Ratio), SSIM (Structual Similarity) [39], and FSIMc (Feature-Similarity-Color) [40] values. The CPSNR measures the ratio of the power of the signal to the power of the corrupting noise, and serves as a measure of the level of the noise, whereas the SSIM and the FSIMc evaluate the similarities of the structure and the feature between two images, respectively.

We performed demosaicing on the Bayer CFA with the residual interpolation(RI) [14], the adaptive residual interpolation(ARI) [15], the inter-color correlation(ICC) [12], and the deep learning network(DNet) [13] based demosaicing methods. For the RGBW CFAs, we compared with the demosaicing method developed by the Sony corporation [27] and the Paul’s method [36].

**Figure 4:**
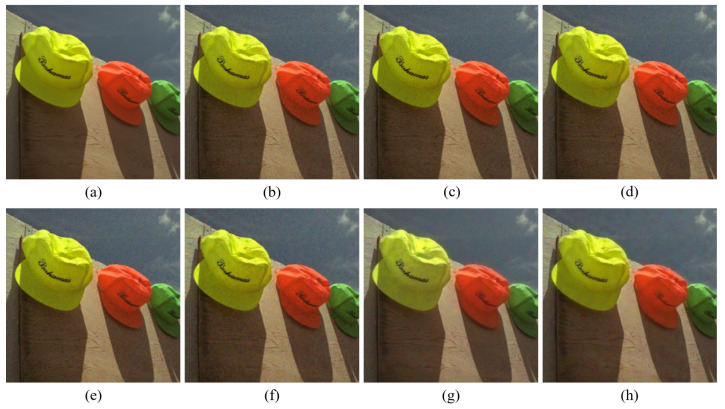
Demosaicing results of the Kodak No. 3 image under low-noise condition. (**a**) Original (**b**) ICC [12], (**c**) DNet [13] (**d**) RI [14] (**e**) ARI [15] (**f**) Sony [27] (**g**) Paul’s [36] (**h**) Proposed.

**Figure 5:**
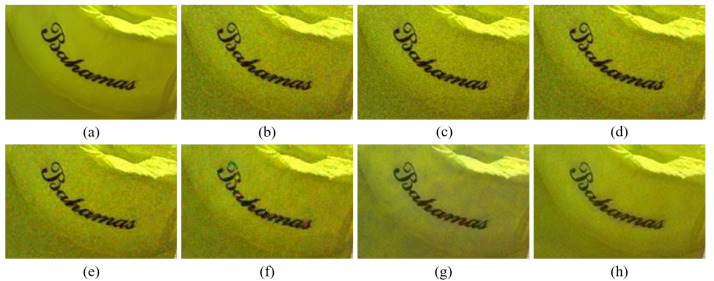
Showing the enlarged regions of Figure 4. (**a**) Original (**b**) ICC [12], (**c**) DNet [13] (**d**) RI [14] (**e**) ARI [15] (**f**) Sony [27] (**g**) Paul’s [36] (**h**) Proposed.

**Figure 6:**
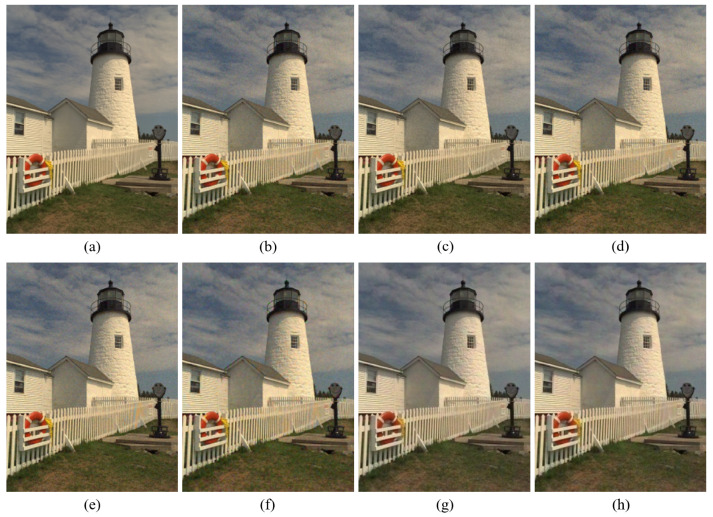
Demosaicing results of the Kodak No. 19 image under low-noise condition. (**a**) Original (**b**) ICC [12], (**c**) DNet [13] (**d**) RI [14] (**e**) ARI [15] (**f**) Sony [27] (**g**) Paul’s [36] (**h**) Proposed.

**Figure 7:**
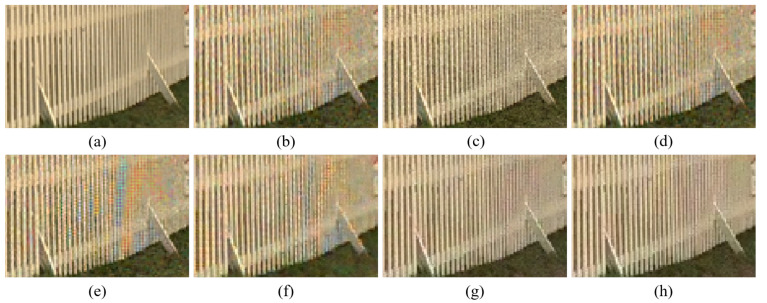
Showing the enlarged regions of Figure 6. (**a**) Original (**b**) ICC [12], (**c**) DNet [13] (**d**) RI [14] (**e**) ARI [15] (**f**) Sony [27] (**g**) Paul’s [36] (**h**) Proposed.

**Figure 8:**
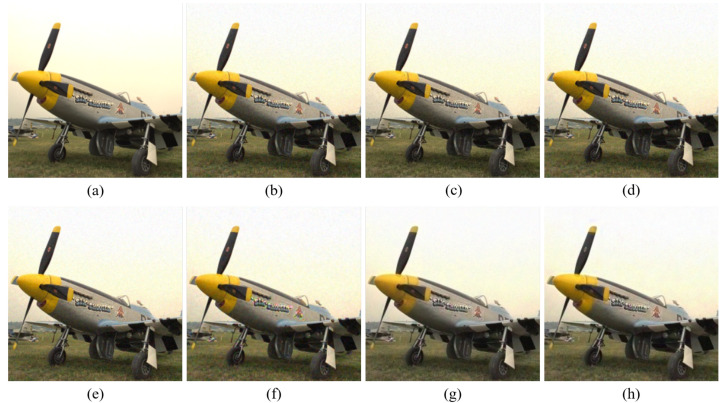
Demosaicing results of the Kodak No. 20 image under low-noise condition. (**a**) Original (**b**) ICC [12], (**c**) DNet [13] (**d**) RI [14] (**e**) ARI [15] (**f**) Sony [27] (**g**) Paul’s [36] (**h**) Proposed.

**Figure 9:**
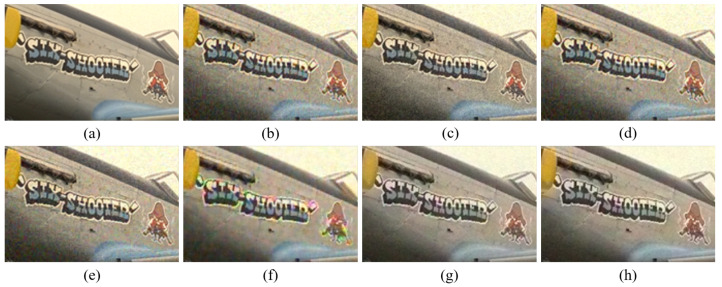
Showing the enlarged regions of Figure 8. (**a**) Original (**b**) ICC [12], (**c**) DNet [13] (**d**) RI [14] (**e**) ARI [15] (**f**) Sony [27] (**g**) Paul’s [36] (**h**) Proposed.

Figure 4, Figure 5, Figure 6, Figure 7, Figure 8 and Figure 9 show the demosaicing results for the low-noise case on the Kodak No. 3, Kodak No. 19, and Kodak No. 20 images. The noise in the Bayer and the Sony CFAs results in high remaining noise, as can be seen in Figure 4a–f, Figure 5a–f, Figure 6a–f, Figure 7a–f and Figure 8a–f. Furthermore, there are some color artifacts, as can be especially observed in the enlarged images in Figure 6a–f and Figure 8a–f. This is because the local interpolation is locally affected by the local noise, which results in differences in the reconstructed colors. Using the Sony RGBW CFA, the RGBW format is first converted to the Bayer RGB format with the Sony demosaicing method [27], which results in additional color aliasing artifacts. Therefore, even though the reconstructed color image has an overall sensitivity improvement because of the higher sensitivity of W pixels, the spatial resolution degradation and the aliasing result in some false colors which are visually unpleasant. This kind of false color artifacts is typical for RGBW CFAs. However, using the Paul’s method [36] and the proposed method on the W-dominant RGBW CFA, the color artifacts are reduced as both the Paul’s method and the proposed method first reconstruct the W channel from the 75% white pixels, and then use it as a guidance for reconstructing the colors. The reconstructed W channel suffers less from the noise than the RGB channels, as it has higher sensitivity.

Therefore, the reconstruction of the colors under the guidance of the W channel also becomes less prone to the noise, which is the reason that the reconstructed color channels have less color artifacts. As can be seen in Figure 6g,h, Figure 7g,h and Figure 8g,h, the results of the proposed method and the Paul’s method are visually similar. Table 1 shows the CPSNR, the SSIM, and the FSIMc values of the various demosaicing results on the Kodak and the McMaster datasets. The bold texts in the tables represent the largest CPSNR, SSIM, and FSIMc values for the various demosaicing methods. The proposed method shows the largest CPSNR and SSIM values in both the Kodak and the McMaster datasets, which indicates the fact that the proposed method reconstructs a color image with the least noise and well preserved structure of the image. The Paul’s method and the proposed method show larger FSIMc values than other demosaicing methods, which indicates the fact that the features in the reconstructed images are better preserved than with other methods.

However, using the Paul’s method, there are some deteriorations in the color when a certain color channel has low intensity values, as can be seen in Figure 4g and Figure 5g. This artifact is due to the noise added to the color seeds. Normally, the noise in the color seeds will cancel each other out by the diffusion in the colorization process. However, when the original color channel has intensity values close to zero, the Paul’s method fails to reconstruct the zero-like intensity values owing to the noise. This is because the sensors always receive positive light energies; thus, the effect of the noise cannot be compensated by negative values, as the the negative values are clamped to zero by the sensors. Therefore, many color seeds that should have values close to zero have positive values much larger than zero, and the diffusion of these values results in false colors. This can be seen as a type of low-frequency noise, which is common in colorization methods.

**Figure 10:**
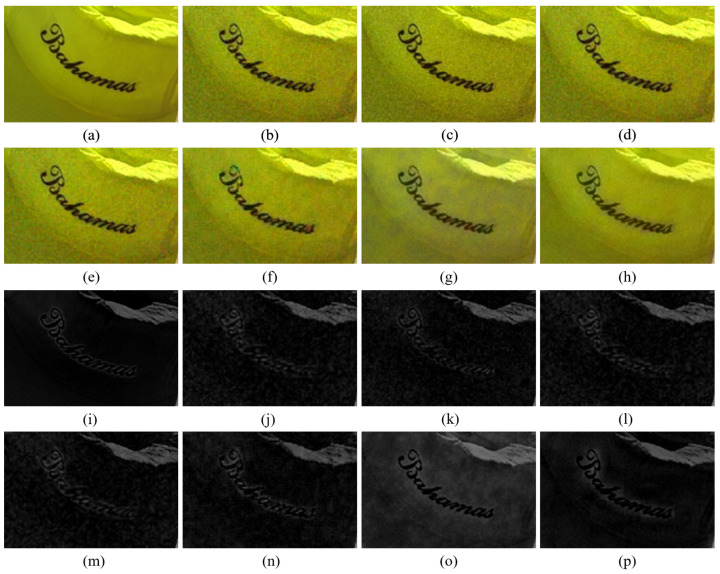
Demosaicing results when one channel has low intensity values. The first and the second rows show the reconstructed color images, while the third and the fourth rows show the Blue channels. (**a**) Original (**b**) ICC [12], (**c**) DNet [13] (**d**) RI [14] (**e**) ARI [15] (**f**) Sony [27] (**g**) Paul’s [36] (**h**) Proposed (**i**) Original (**j**) ICC [12] (**k**) DNet [13] (**l**) RI [14] (**m**) ARI [15] (**n**) Sony [27] (**o**) Paul’s [36] (**p**) Proposed.

**Figure 11:**
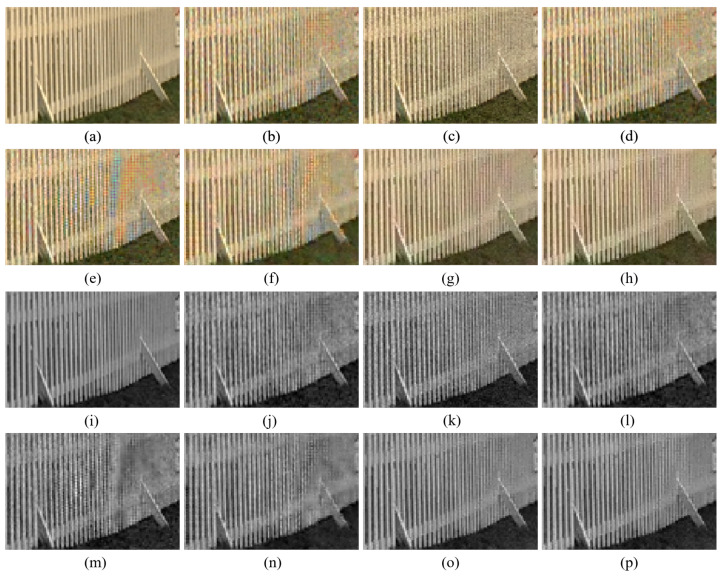
Demosaicing results when none of the channels has low intensity values. The first and the second rows show the reconstructed color images, while the third and the fourth rows show the Blue channels. (**a**) Original (**b**) ICC [12], (**c**) DNet [13] (**d**) RI [14] (**e**) ARI [15] (**f**) Sony [27] (**g**) Paul’s [36] (**h**) Proposed (**i**) Original (**j**) ICC [12] (**k**) DNet [13] (**l**) RI [14] (**m**) ARI [15] (**n**) Sony [27] (**o**) Paul’s [36] (**p**) Proposed.

Figure 10 shows the case where the B channel in the original color image has intensity values that are close to zero. Even though the noise is smaller than in the color channels reconstructed by other demosaicing methods, it can be seen in Figure 10g,o that the reconstructed B channel has large intensity values, where it actually should have small values. This results in deterioration of the reconstructed color. This type of artifact is reduced with the proposed method, owing to the rank minimization, as can be seen in Figure 10h,p. The rank minimization smooths out the noise in the color seeds and keeps the intensity values low. This is due to the fact that a majority of the intensity values are low and the low-rank structure follows the trend of the majority. Therefore, the proposed method reconstructs the colors better than the Paul’s method. Meanwhile, Figure 11 shows the case, where all the color channels in the original image have values much larger than zero. In this case, both the Paul’s method and the proposed method can well reconstruct the colors.

**Figure 12:**
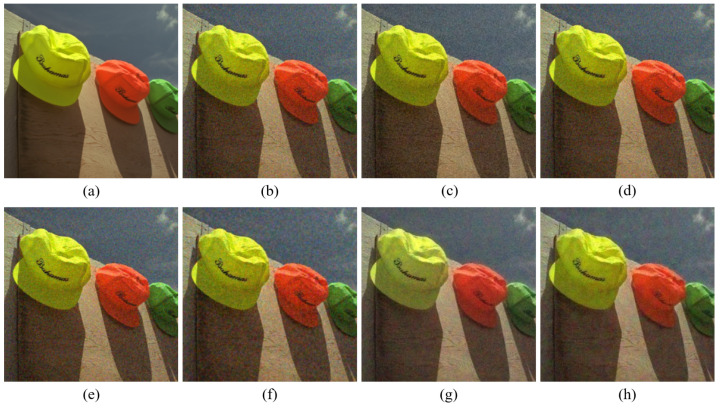
Demosaicing results of the Kodak No. 3 image under high-noise condition. (**a**) Original (**b**) ICC [12], (**c**) DNet [13] (**d**) RI [14] (**e**) ARI [15] (**f**) Sony [27] (**g**) Paul’s [36] (**h**) Proposed.

**Figure 13:**
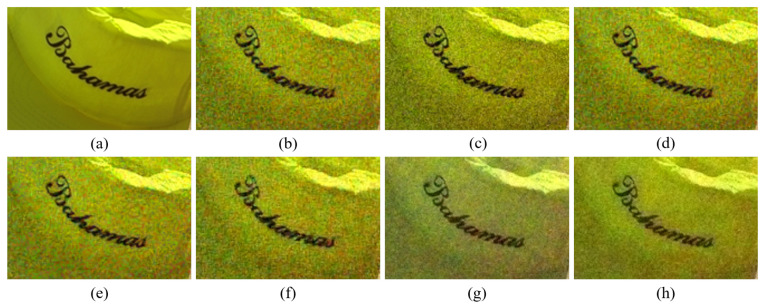
Showing the enlarged regions of Figure 12. (**a**) Original (**b**) ICC [12], (**c**) DNet [13] (**d**) RI [14] (**e**) ARI [15] (**f**) Sony [27] (**g**) Paul’s [36] (**h**) Proposed.

**Figure 14:**
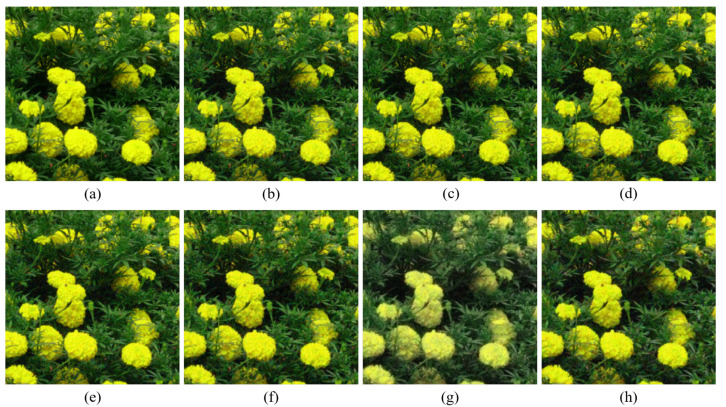
Demosaicing results of the McMaster No. 16 image under high-noise condition. (**a**) Original (**b**) ICC [12], (**c**) DNet [13] (**d**) RI [14] (**e**) ARI [15] (**f**) Sony [27] (**g**) Paul’s [36] (**h**) Proposed.

**Figure 15:**
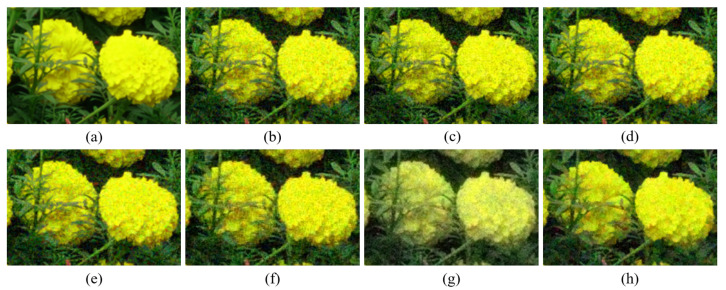
Showing the enlarged regions of Figure 14. (**a**) Original (**b**) ICC [12], (**c**) DNet [13] (**d**) RI [14] (**e**) ARI [15] (**f**) Sony [27] (**g**) Paul’s [36] (**h**) Proposed.

**Figure 16:**
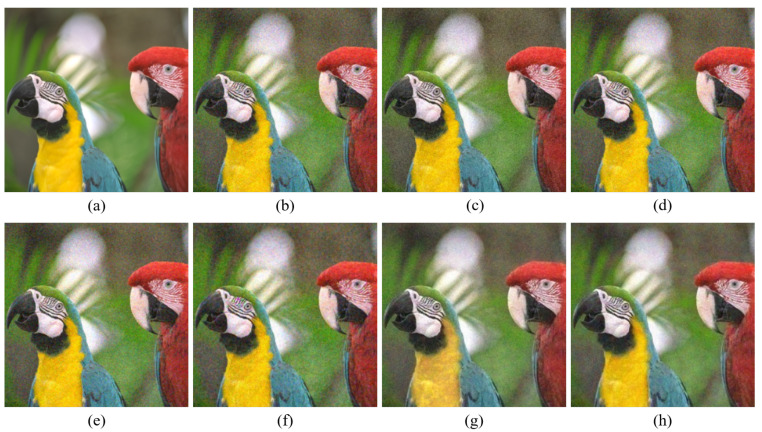
Demosaicing results of the Kodak No. 23 image under high-noise condition. (**a**) Original (**b**) ICC [12], (**c**) DNet [13] (**d**) RI [14] (**e**) ARI [15] (**f**) Sony [27] (**g**) Paul’s [36] (**h**) Proposed.

**Figure 17:**
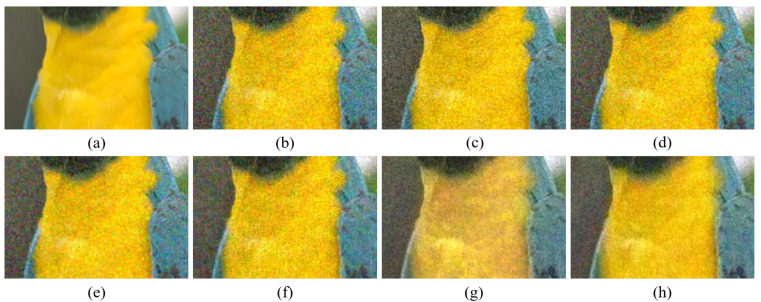
Showing the enlarged regions of Figure 16. (**a**) Original (**b**) ICC [12], (**c**) DNet [13] (**d**) RI [14] (**e**) ARI [15] (**f**) Sony [27] (**g**) Paul’s [36] (**h**) Proposed.

Figure 12, Figure 13, Figure 14, Figure 15, Figure 16 and Figure 17 and Table 2 show the results for the high-noise case. In the high-noise case, the noise is derived from a zero-mean Gaussian distribution with standard deviations of 0.1463, 0.0929, 0.1018, and 0.0496 for the R, G, B, and W channels, respectively. The proposed method shows the largest average CPSNR and SSIM values for all datasets, again demonstrating the fact that the proposed method is the most robust one against the noise and preserves well the structures of the image. Furthermore, it can be observed that the Paul’s method intensifies the problem of deteriorated colors as the noise becomes larger. However, using the proposed method the colors are well reconstructed.

## 5. Conclusions

In this paper, we proposed a rank minimization-based matrix completion method with a colorization-based constraint for the demosaicing of the white-dominant color filter array (CFA). The matrix completion performs a structured global interpolation, while the colorization-based constraint evaluation performs a local interpolation and preserves the colors. Both processes mutually compensate for the weaknesses of each other, i.e., the matrix completion helps to maintain the global structure and eliminates local artifacts, whereas the colorization-based constraint helps to overcome the over-smoothing problem in the global interpolation and preserve the colors.

Therefore, we proposed a demosaicing method that is more robust against noise than other demosaicing methods. The proposed method can be used for surveillance camera applications, as surveillance cameras have to capture images in low illumination environments and the CFA image becomes noisy, owing to the large ratio of light energy versus thermal noise. In the experiments, we used Kodak and McMaster datasets, and compared the robustness of different demosaicing methods against noise in terms of the CPSNR, SSIM, and the FSIMc measures. The proposed method achieved CPSNR values that were approximately 1.5 dB greater than those of RGB CFA based demosaicing methods such as the residual interpolation (RI) [14], adaptive residual interpolation (ARI) [15], inter-color correlation (ICC) [12], and even a deep learning based method (DNet) [13]; thus, we verified the robustness of our proposed method against noise. Compared with RGBW based demosaicing methods, the proposed method achieves CPSNR values approximately 0.5 dB and 0.3 dB greater than those of Sony’s [27] and Paul’s [36] methods, respectively. Furthermore, when compared to the Paul’s method, the proposed method can overcome the problem of deteriorated colors in regions with low R, G, or B intensity values.

The proposed method uses a rank minimization with respect to the DCT basis. Further studies can elaborate the usage of other bases apart from the DCT basis. For example, if an optimal basis is learned from the image, it can lead to performance improvement. Besides, a study on sophisticated methods that can combine the global and the local interpolation constraints more effectively can be another topic for further studies.

## Figures and Tables

**Table 1:** Comparison of the CPSNR, SSIM, and FSIMc values between the various demosaicing methods on the Kodak, the McMaster, and the Kodak+McMaster image datasets under the low-noise condition. The bold texts represent the largest values.

Measure	Dataset	Methods						
		ICC [12]	DNet [13]	RI [14]	ARI [15]	Sony [27]	Paul’s [36]	Proposed
CPSNR	Kodak	28.77	28.79	28.69	29.00	29.17	30.72	**30.98**
	McMaster	28.83	28.88	28.59	28.99	28.80	27.79	**29.66**
	Kodak+McMaster	28.80	28.83	28.64	28.99	29.01	29.47	**30.41**
SSIM	Kodak	0.8471	0.8487	0.8453	0.8543	0.8900	0.9235	**0.9257**
	McMaster	0.8840	0.8892	0.8803	0.8891	0.9091	0.9039	**0.9176**
	Kodak+McMaster	0.8629	0.8661	0.8603	0.8692	0.8981	0.9151	**0.9223**
FSIMc	Kodak	0.9521	0.9540	0.9517	0.9552	0.9615	**0.9794**	0.9774
	McMaster	0.9571	0.9579	0.9563	0.9599	0.9641	**0.9715**	0.9705
	Kodak+McMaster	0.9542	0.9557	0.9537	0.9572	0.9626	**0.9760**	0.9745

**Table 2:** Comparison of the CPSNR, SSIM, and FSIMc values between the various demosaicing methods on the Kodak, the McMaster, and the Kodak+McMaster image datasets under the high-noise condition. The bold texts represent the largest values.

Measure	Dataset	Methods						
		ICC [12]	DNet [13]	RI [14]	ARI [15]	Sony [27]	Paul’s [36]	Proposed
CPSNR	Kodak	23.42	22.87	23.37	23.69	24.45	25.28	**25.35**
	McMaster	23.81	23.50	23.65	24.03	24.23	23.73	**24.49**
	Kodak+McMaster	23.59	23.14	23.49	23.83	24.35	24.62	**24.98**
SSIM	Kodak	0.6543	0.6344	0.6522	0.6675	0.7103	0.7256	**0.7282**
	McMaster	0.7507	0.7477	0.7466	0.7602	0.7767	0.7574	**0.7727**
	Kodak+McMaster	0.6956	0.6830	0.6926	0.7072	0.7387	0.7392	**0.7473**
FSIMc	Kodak	0.8699	0.8733	0.8696	0.8785	0.8845	**0.9178**	0.9147
	McMaster	0.8877	0.8896	0.8869	0.8950	0.8951	**0.9180**	0.9138
	Kodak+McMaster	0.8775	0.8803	0.8770	0.8855	0.8890	**0.9179**	0.9143
